# The Potential Correlation Between Bacterial Sporulation and the Characteristic Flavor of Chinese Maotai Liquor

**DOI:** 10.3389/fmicb.2018.01435

**Published:** 2018-07-02

**Authors:** Weiying Wang, Renlu Liu, Ye Shen, Bin Lian

**Affiliations:** ^1^Jiangsu Key Laboratory for Microbes and Functional Genomics, College of Life Sciences, Nanjing Normal University, Nanjing, China; ^2^College of Life Sciences, Jiangxi Normal University, Nanchang, China; ^3^Shanghai OE Biotech. Co., Ltd., Shanghai, China

**Keywords:** Maotai-flavor substances, high-temperature fermentation, *Bacillus subtilis*, sporulation, transcriptome, microarray

## Abstract

The relationship between the formation of characteristic Maotai-flavor substances (MTFS) and the dominant bacteria in Maotai Daqu (MTDQ) has long been a topic of research interest in the field of liquor brewing in China. To investigate the connection between MTFS and the *Bacillus subtilis* (one of dominant bacteria in MTDQ) cultured on solid plates of wheat extract medium at, temperatures of 37, 46, and up to 55°C (Group A), and at a constant 37°C (Group B), the transcriptomes of the bacteria grown in the two groups were studied. About 10 out of 84 differentially expressed genes (DEGs) were related to promoting sporulation. Furthermore, observations made with transmission electron microscopy (TEM) showed that a thicker spore cortex appeared in Group A. The content of 2, 6-pyridinedicarboxylic acid (DPA), an important component of the spore, was 49.77 (±2.50) and 38.23 (±3.96) μg/mg of dried spores from the bacteria cultured in Groups A and B, respectively. Combined with the production process of Maotai liquor, more DPA accumulates in the high-temperature fermentation stage and is then released by spore germination during the subsequent temperature-drop stage. We suggest that DPA (or its derivatives) can then be transformed into MTFS by the Maillard reaction after many rounds of microbial fermentation. The viewpoint that there is a potential correlation between bacterial sporulation and the production of MTFS is proposed.

## Introduction

The main components of liquor are ethanol and water. These two compounds account for more than ∼98% of the liquor. The quality of the liquor mostly depends on a variety of scented organic compounds which account for less than 2% (Wu and Liu, 2005). Maotai liquor, made in Maotai town in the Guizhou Province in China, is the classic representative of Maotai-flavor style liquors. It is a type of natural product made via microbial solid fermentation without adding any other flavoring substances (Fan et al., 2011; Wu et al., 2013). The characteristic Maotai-flavor substances (MTFS) that are present in Maotai liquor have been identified to be a collection of small molecular compounds and include pyridines, pyrazines, organic acids, esters, alcohols, aldehydes, ketones, and other aromatic compounds (Azokpota et al., 2010; Chen et al., 2013; Lin et al., 2013). However, where exactly the Maotai flavor comes from, and what the relationship is between the MTFS and the fermenting microbes, remain unclear. Because Maotai has a superior position in China’s liquor market, its constitution and the mechanism of formation of the MTFS have always been hot topics of research in liquor brewing (Zhou, 1983; Xu et al., 2011).

Maotai Daqu (MTDQ) used in the production of Maotai liquor, which is mainly made using wheat as the raw material, goes through several rounds of microbial fermentation from low to high temperature (Wang et al., 2008; Zheng et al., 2011; Liu et al., 2012) and plays an essential role in the Maotai-flavor liquor brewing process. MTDQ, which is rich in a variety of microorganisms and enzymes, is essential for making Maotai liquor. In actual production, the dosage of MTDQ is large, which is almost half of the total mass of liquor-making raw material. MTDQ is not only a component of the raw materials, but is also involved in the formation of ethanol and aroma compounds (Lian, 1997; Lin et al., 2013). The specific production environment and stable production technology make MTDQ not only form a relatively stable microbial ecosystem, but also form the unique flavor base of Maotai liquor. It is known that heat-resistant bacteria are the main driving force in the MTDQ fermentation process (Xiong, 2005). Lian (1997) pointed out that the microbial action and the Maillard reaction in the process of MTDQ manufacture and the liquor brewing process are the reasons for all the flavor substances produced. However, the Maotai-style flavor components derived from the microbial activity in MTDQ remain an intriguing mystery in this field.

The special Maotai liquor brewing process includes high-temperature starter-making, high-temperature stacking, and high-temperature fermentation, all of which are essential conditions for producing Maotai-flavor liquor (Wang et al., 2008). The MTDQ processing temperature is usually up to and over 55°C, otherwise the MTDQ produces poor MTFS (Yang et al., 2004). The isolates from MTDQ include almost exclusively *Bacillus* spp., especially *Bacillus subtilis, B. licheniformis*, and *B. amyloliquefaciens* (Li et al., 2014), which are commonly viewed by most researchers as those components that contribute most to flavor production during MTDQ fermentation (Lian et al., 1993; Luo and Xie, 2012). *B. subtilis* is one of the dominant bacteria in MTDQ during the high-temperature fermentation stage, and the formation of MTFS requires this process of high-temperature fermentation. This, logically and naturally, makes us think there is a potential link between the *Bacillus* spp., high-temperature fermentation, and MTFS formation. Therefore, it is necessary to investigate the connection between metabolites produced from *B. subtilis* cultured at high temperatures and MTFS formation; however, this is difficult to understand using traditional analytical techniques and methods.

Modern techniques in molecular biology have been adopted to analyze the change in gene expression on a molecular level so as to expound the change in gene expression response to different environmental conditions (Cho and Tiedje, 2002). In this context, investigating the gene expression profile of *B. subtilis* under high-temperature fermentation using microarrays may help us to understand the mechanisms underlying flavor-production caused by the organisms during the high-temperature stage. Presently, there is a lack of in-depth research directly relating genes and their involvement in the metabolic pathways producing flavor. Wu and Xu (2012) attempted to clarify the mechanism of MTFS production by analyzing the gene expression profiles of *B. licheniformis* and their response to high-temperature stress using microarrays. They found a number of important differentially expressed genes (DEGs), most of which encode heat shock proteins which can help bacteria to survive at high temperatures, and also found that cysteine may be used as precursor to the synthesis of MTFS. Further research is needed to elucidate the molecular mechanism of MTFS formation.

To explore the differences in bacterial metabolites and gene expression at different culture temperatures, considering microbial fermentation of wheat caused gradual warming in the manufacturing process of Maotai-Daqu, a gradual warming mode of 37–46–55°C was designed to represent the high-temperature fermentation process used there. A strain of the dominant *B. subtilis* isolated from MTDQ was used as the research subject here. The gene expression profiles of bacteria cultured on solid plates made from wheat extract from low to high temperature (37–46–55°C) were analyzed and compared with those from bacteria cultured at a constant 37°C. The response of the related genes and pathways to the high-temperature stress was obtained so as to analyze the mechanism of MTFS production. Thus, we provide basic data for further understanding the origin of the flavor of Maotai liquor and for effectively utilizing this special fermented bacterial-resource.

## Materials and Methods

### Strain and Culture

The strain used in this experiment was isolated from MTDQ (Li et al., 2014). It was identified as *B. subtili*s via 16SrDNA analysis (GenBank database accession number: KT343640) and Biolog instrumentation (PROB = 0.610, SIM = 0.610, DIST = 5.714). It is one of the dominant bacteria in MTDQ and is considered to be one of the MTFS-producing functional bacteria (Lian et al., 1993; Lian, 1997; Lin et al., 2013; Li et al., 2014).

To acquire the seed liquid, *B. subtilis* was inoculated into 100 mL of Luria–Bertani liquid medium and cultured overnight at 37°C and 200 rpm. Wheat extract combined with beef–protein (WEBP) was then used as a test medium to acquire the total bacterial RNA. The medium was prepared as follows: Wheat (150 g) was added to deionized water (1 L) and left overnight at 37°C. After boiling for 60 min, the filtrate passing through four-layer gauze was collected. 0.75 g of beef extract, 2.5 g of peptone and 1.25 g of sodium chloride (a quarter amount of beef extract peptone medium) and agar (20 g) were dissolved into the filtrate (supplementing deionized water up to 1000 mL) and the mixture sterilized in an autoclave. With gentle shaking and pouring when the medium cooled down to about 55°C, plates were formed. The experiment was divided into two distinct groups, as follows:

Group A (high temperature): Seed liquid (200 μL) was spread evenly over the plate of the WEBP medium, and then was inversion cultured at 37°C for 12 h, then cultured at 46°C for 12 h, and finally at 55°C for 24 h.

Group B (low temperature): The method is the same as in Group A except that the culture temperature was kept at 37°C only (48 h).

The *B. subtilis* samples in plates were taken at 12, 24, and 48 h for the counting of spores, respectively. The experiment was conducted as follows: first, the bacteria were diluted into OD_600_ = 1.0, and then incubated at 75°C for 15 min to kill any vegetative cells. Finally the bacteria, with the appropriate dilution, were spread on agar plates of beef extract peptone for spore germination and the count of bacterial colonies in plate multiplied by the dilution factor gave the amount of initial spores per OD_600_.

At the end of the cultivation period, the bacterial culture on the plates was collected from the two groups and quickly transferred, respectively, to a 1.5 mL centrifuge tube containing lysozyme solution (300 μL) made using RNase-free water. It was then placed immediately into liquid nitrogen for 10 min and stored at -80°C until total RNA extraction was conducted.

At the same time, some of the bacterial culture from the two groups was rapidly taken for observation using TEM. For this, the samples were fixed using 2% glutaraldehyde and 1% osmium tetraoxide. Then, they were embedded in an epoxy resin and sectioned according to the method of Kay and Warren (1968) for TEM observation. The thickness of spore cortex in the field of view of a microscope was measured by the ruler tools in the Adobe Photoshop CS5 program. The data analysis was conducted by Independent-sample *T*-test in SPSS program.

### Total RNA Extraction

High-quality total RNA of *B. subtilis* under high-temperature conditions cannot be obtained by using several bacterial RNA extraction kits, so a modified cetyl trimethyl ammonium bromide (CTAB) method was used for total RNA extraction from the two groups of *B. subtilis*. The composition of the CTAB extraction solution were as follows: 2% CTAB, 30 mM EDTA, 2 M NaCl, 100 mM Tris-HCl and 0.05% spermidine. The final solution was adjusted to a pH of 8.

First, for the cell lysis stage, a lysis solution was prepared by adding 600 μL of CTAB extraction solution, 12 μL of 2% polyvinylpyrrolidone (PVP), 12 μL of beta-mercaptoethanol, 1.5 mg/mL (final concentration) protease K and 100 μL of lysozyme (1 mg/mL) to a 1.5 mL undefiled centrifugation tube without RNase. The lysis solution was incubated for 10 min at 42°C and then transferred to a specimen tube and incubated for 90 min at 42°C. The sample tubes were swirled gently several times during the whole time.

Next, RNA was separated and purified using the following method. Chloroform–isoamyl alcohol extraction solution was added to the sample tubes and mixed upside-down, and then they were centrifuged for 15 min at 13,000 rpm at 4°C. The upper, aqueous layer was carefully removed to another clean centrifuge tube and treated again using the same procedure. A pre-cooled solution containing an equal volume of LiCl (4 M) and half the volume of ethanol was used to precipitate RNA from the aqueous solution from the above steps. This was put on ice for 3 h to obtain a pellet. Then, the resulting pellet was washed with 600–800 μL of 2 M LiCl and 75% ethanol, followed by 5 min of centrifuging at 13,000 rpm, 4°C. The dried pellet was ultimately dissolved using an appropriate amount of water treated with diethylpyrocarbonate to obtain the bacterial total RNA.

Total RNA was quantified using a UV-vis spectrophotometer (ND-2000, Thermo Scientific) and RNA integrity was determined using a bioanalyzer (Agilent 2100, (Agilent Technologies).

### *c*DNA Synthesis and Microarray Hybridization

After obtaining the qualified RNA, cDNA was synthesized using AffinityScript HC reverse transcriptase (Takara) with a mixture of dNTP with amino-allyl dUTP, random primer, and 5 μg total RNA. Then, 1 M NaOH was added and the mixture was incubated for 10 min at 70°C to hydrolyze the RNA. Fluorescently labeled cDNA was obtained by adding 5 μL of Cy3 Mono-Reactive Dye to the cDNA (which binds to the amino-allyl dUTP). Finally, the cDNA was hybridized with the customized microarray (Agilent-066044) for 17 h at 65°C. Fluorescence was measured by scanning the microarray with a microarray scanner (Agilent G2505C), and the DEGs were elucidated by comparing the fluorescence intensity from probes in the two groups. The total RNA extraction/hybridization procedure was performed in quadruplicate.

### Analysis of the Differentially Expressed Genes

To begin with, the raw data were normalized using the quantile algorithm. The probes that had at least 100% of the values in any one out of all conditions flagged as ‘detected’ were chosen for further data analysis. DEGs were then identified through their fold change as well as the *P*-value calculated by using a *t*-test. The threshold set for up- and down-regulated genes was an *n*-fold change of *n* ≥ 2.0 and a *P* ≤ 0.05. In this research, all the up- and down-regulated expression are the relative quantitative expression of gene in Group A compared to Group B. To analyze the molecular functions of the DEGs, and the relevant metabolic pathways, the DEGs were annotated using the GO (Harris et al., 2004) and KEGG (Kanehisa et al., 2008) databases, followed by enrichment of the terms (*P* < 0.05 for significant enrichment). The three child databases of GO — cellular component (CC), biology process (BP), and molecular function (MF) — were adopted to analyze the cellular localizations and functional classifications of the DEGs. Finally, hierarchical clustering was performed to display the distinguishable genes’ expression pattern among the samples. GeneSpring software was employed to finish the basic analysis on the raw data (Wu and Xu, 2012).

### Quantitative Real Time RT-PCR (QPCR)

Quantification was performed using a two-step reaction process: reverse transcription (RT) and PCR. PrimerScript RT Enzyme Mix I (TaKaRa, Japan) reverse transcriptase was employed to prepare cDNA from amplified RNA. With 16srRNA as the reference gene, the 16srRNA primers used were forward-5′GTGGACTACCAGGGTATCTAAT3′ and Reverse-5′ GGTGAAATGCGTAGAGATGTG3′. The relative transcript abundance of the genes was detected using SYBR Green (Roche, Switzerland) and a real-time PCR thermal-cycler instrument (LightCycler^®^ 480 II, Roche, Switzerland) using gene-specific primers. The specificity of the primer pairs was as follows: *cspB*, 5′-TCGAAGTAGAAGGTCAAGACG-3′ (forward) and 5′-GCGGTTTCCTTCAACGATT-3′ (reverse); *gerPF*, 5′- CGGAGTCGTAAACTTTGGT-3′ (forward) and 5′-CCATATCTTGATCGCTGACAT-3′ (reverse). Each sample was run in quadruplicate in this analysis.

### Assay for DPA in Bacillus Spores

The culture conditions for Group A and B were the same as those described in Section “Strain and Culture.” The culture, in plates, was scraped into a clean centrifuge tube, then washed and centrifuged with decreasing rotor speeds (8,200, 4,200, and 2,700 g for 15 min at 4°C). The resulting spore pellet, after each centrifugation, was suspended in ice-cold distilled water. Spores were then pasteurized for 15 min at 70°C to kill vegetative cells, and then washed and centrifuged with decreasing rotor speeds (4,200 g, then 2,700 g for 15 min at 4°C) (Gounina-Allouane et al., 2008). Assay of DPA in bacillus spores was undertaken using procedures described previously (Janssen et al., 1958; Rotman and Fields, 1968; Planchon et al., 2011). DPA was extracted from the spores by autoclaving at 121°C for 30 min and then cooling. Autoclaved suspensions were centrifuged at 7,000 *g* for 15 min at room temperature. A 2 ml volume of each sample was mixed with 0.5 ml of reagent composed of 1% ammonium iron (II) sulfate [Fe(NH_4_)_2_(SO_4_)_2_⋅6H_2_O] and 0.1% cystein in sodium acetate buffer 0.05 M, at pH 4.0. Then, the optical density was measured at 400 nm. The DPA concentration was obtained from a calibration curve using a 1 mg/ml DPA (LookChem, Shanghai, China) solution. Spore DPA content was expressed in μg per mg mass of dried spores. The data analysis was conducted by using the independent-sample *T*-test in the SPSS program.

### Gas Chromatography–Mass Spectrometry (GC-MS) of the Fermentation Products

To simulate solid fermentation of wheat in the Maotai brewery, the cleansed wheat was autoclaved after being soaked overnight in deionized water at 37°C. An appropriate amount of water was added to the wheat to bring its moisture content to around 55%, at which, the wheat materials could be pinched into a ball and loosened into dispersed particles of wheat thereafter. The prepared wheat (100 g) was added to a 250 ml conical flask and sealed with eight-layer gauze, and sterilized in an autoclave. Seed liquid (5 mL) was inoculated into the medium (the aforementioned prepared wheat), stirred with a sterile glass rod, and then placed in incubators for static culture. The culture method involved high-temperature fermentation (as with Group A), and cultivation was conducted over six cycles of the temperature gradient (Group D). The sterile water (5 mL) replacing the bacterial culture was added to the medium to act as a control for Group D (namely, Group E). Group F was the same as Group D but was only cultured at 37°C. The cultivation of bacteria was performed in sextuplicate.

After the cultivation period of 12 d, anhydrous ethanol (100 mL) was added to the fermentation medium in the flasks (just covering the wheat culture) from the three groups, respectively, and shocked at 220 rpm for 3 h. Each duplicate was merged into one group. The extracts from the same groups were concentrated using a rotary evaporator and dried over anhydrous sodium. The samples were analyzed by GC-MS after dilution and the operational program and conditions followed a published method (Li et al., 2014) except that the scan mass spectra within the scan range of 50 to 650 amu were assessed. Identification of individual compounds was made possible by comparison of their mass spectra and retention indices with those of the internal reference mass spectra library NIST05.

## Results

### Morphology of *B. subtilis*

**FIGURE 1:**
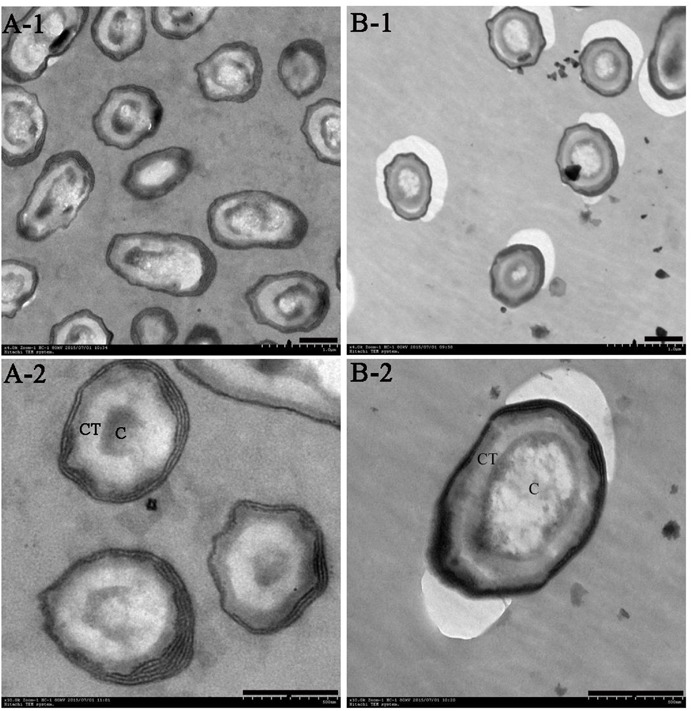
Transmission electron microscopy (TEM) of *B. subtilis* after cultivation for 48 h in WEBP medium. **(A-1, A-2)** Show the morphology of *B. subtilis* from Group A magnified 4,000 times and 10,000 times, respectively. **(B-1, B-2)** Indicate the morphology of *B. subtilis* from Group B magnified 4,000 times and 10,000 times, respectively. CT, spore cortex; C, spore core (the scale bars are all 0.5 microns in length).

After fermenting the *B. subtilis* for 48 h on WEBP solid plates, the Group A plate appeared brown while the Group B plate remained a shallow khaki color. The Group B sample formed a thicker bacterial layer on the surface of the plate compared to the Group A one. The two cultures emitted different odors and the bacterial cell morphologies obtained by TEM are shown in **Figure 1**.

The population of spores increased gradually with the increase in temperature in Group A, and there were significant differences in the number of spores at three different temperatures. Although the incubation temperature of bacteria in Group B was unchanged, the spores were already evident at 12 h, and there was no significant difference in the number of spores at 12, 24, and 48 h, however, at the end of the culture period (48 h), the number of spores in Group A was significantly higher than that in Group B (**Supplementary Figure S1**). Moreover, the spore cortices of the bacteria in Group A were clearly thicker than those in Group B; the former’s nuclei were compact, while the latter’s were evenly dispersed.

### Analysis of the Transcriptome of *B. subtilis* Under Different Culture Conditions DEGs of *B. subtilis* for the Two Different Culture Conditions

The relationship between gene expression in *B. subtilis* in response to high temperature and formation of MTFS was investigated by microarray. The complete set of microarray data has been deposited in the Gene Expression Omnibus (GEO) database at the National Center for Biotechnology Information^1^ (accession number GSE71067).

**FIGURE 2:**
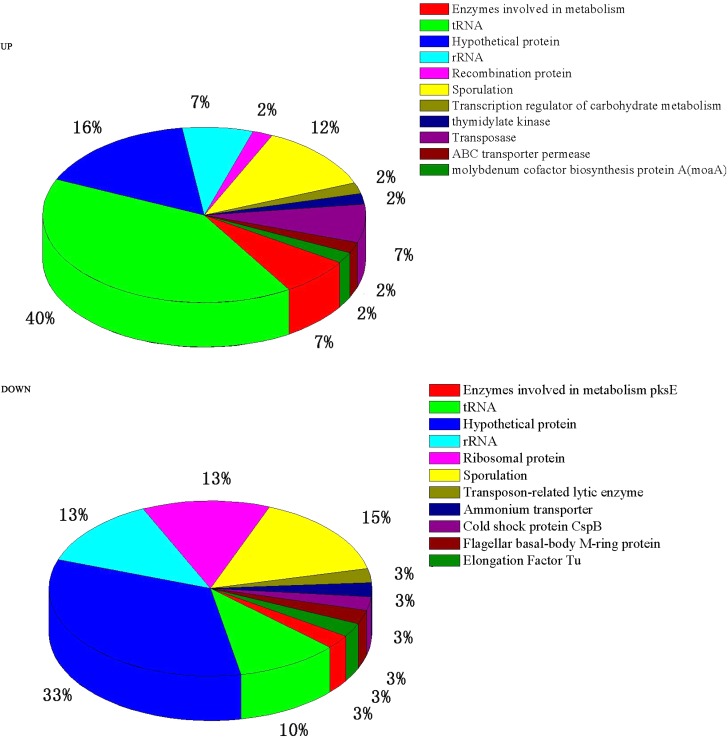
Pie charts showing the function classification of the DEGs.

A total of 84 genes out of 6,820 (*P* ≤ 0.05 and fold change ≥ 2) were found to have significant expression differences between Group A and Group B (44 up- and 40 down-regulated). These DEGs were annotated and classified (**Figure 2**). A large proportion of the DEGs are hypothetical protein coding-genes. Another portion relate to tRNA coding-genes (accounting for 40% of the up- and 10% of the down-regulated DEGs).

In addition, a considerable number of the DEGs are related to sporulation (see **Table 1**). The most significantly different gene is *ybaN* (polysaccharide deacetylase *pdaB*) among the up-regulated DEGs, which is related to the formation and maturation of spores. Then come *sspA* and *sspC* which were up-regulated by 13 and 9 times. SspA and SspC are kinds of small-molecule acid-soluble spore proteins (SASPs) which can bind non-specifically with DNA. The most significantly different gene among the down-regulated DEGs is *rapA* (by 112 times). RapA protein is a kind of aspartic acid phosphatase which is important for sporulation in the *Bacillus* genus. *CotG, cotY*, and *gerPF* were down-regulated by 25, 5, and 3 times, respectively. These are associated with the formation and assembly of the spore outer protein layer. To confirm the microarray data, we analyzed the relative mRNA levels of the two genes *cspB* and *gerPF* using qRT-PCR. The expression levels of *cspB* and *gerPF* were down-regulated by 4.3 and 2.6 times, respectively, which is in agreement with the microarray results.

**Table 1:** The differentially expressed genes (DEGs) related to sporulation.

Up-regulated genes	Fold change	Down-regulated genes	Fold change
*ybaN*	17.51	*rapA*	112.5
*sspA*	13.22	*cotG*	25.79
*sspC*	9.12	*cotY*	5.49
*Bsn5_00005*	3.12	*gerPF*	3.56
*minD*	2.25	*I653_17610*	20.99

The DEGs involved in the metabolic pathways are few in number. Only the genes of glycine C-acetyltransferase (*kbl*) and bifunctional 3,4-dihydroxy-2-butanone 4-phosphate synthase (*ripA*) were up-regulated in Group A compared to Group B. These catalyze the conversion of glycine and 5-phosphate ribose to aminobutyric acid and dihydroxy butanone phosphoric acid, respectively (the biochemical reactions catalyzed by kbl and ripA are as described below).

$$acetyl-CoA+glycine\overset{Kbl}{\to }CoA+L-2-a\min o-3-oxobu\tan otate$$

$$D-ribulose-5-phosphate+formate\overset{RipA}{\to }L-3,4-dihydroxy-2-bu\tan one-4-phosphate$$

One down-regulated gene involved in the metabolic pathway is polyketide biosynthesis protein (*pksE*). The fold changes for the three genes above are 2.9, 4.2, and 4.7 times, respectively.

#### Clustering Analysis of the DEGs

**FIGURE 3:**
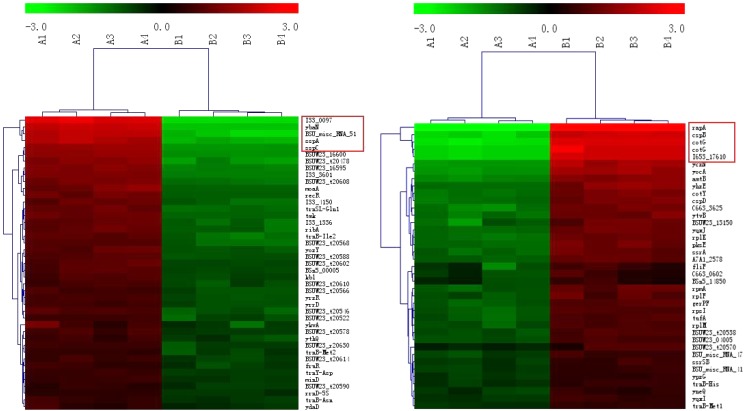
Clustering of the DEGs. The up- and down-regulated genes are on the left and right, respectively. The red boxes represent the genes with the maximum fold changes.

Compared with Group B, the most significantly different genes in Group A (irrespective of up- or down-regulation) are related to sporulation (**Figure 3**, the genes in the red boxes). Examples include *pdaB, sspA, cotG, cotY, etc*. In addition, *cspB*, a gene related to cold-induced shock, was heavily up-regulated in Group B. It was mainly expressed at 37°C in *B. subtilis* (Kaan et al., 1999).

#### Enrichment Analysis of the DEGs

The cellular localization results show that the DEGs are mainly ribosomal components. The function enrichment results show that the DEGs are predominantly responsible for translation and sporulation. Only the ribosome term is significant in the KEGG enrichment analysis (**Table 2**).

**Table 2:** GO and KEGG enrichment analysis of the DEGs.

Gene ontology						KEGG	
	
CC	*P*	BP	*P*	MF	*P*	Pathway	*P*
a	*P* < 0.01	e	*P* < 0.01	h	*P* < 0.01	bsu03010 Ribosome	*P* < 0.01
b	*P* < 0.01	f	*P* < 0.05	i	*P* < 0.01		
c	*P* < 0.01	g	*P* < 0.05				
d	*P* < 0.01						

*a, GO: 0005840 ribosome; b, GO: 0043228 non-membrane-bounded organelle; c, GO:0043232 intracellular non-membrane-bounded organelle; d, GO:0030529 ribonucleoprotein complex; e, GO:0006412translation; f, GO:0043934sporulation; g, GO:0030435sporulation resulting in formation of a cellular spore; h, GO:0003735 structural constituent of ribosome; i, GO:0005198 structural molecule activity.*

### The DPA Content of *Bacillus* Spores Under the Two Different Culture Conditions

The DPA content was significantly higher (*P* < 0.05) in spores from Group A (49.77 ± 2.50 μg per mg of dried spores) than in spores from Group B (38.23 ± 3.96 μg per mg of dried spores).

### Analysis of MTFS in Different Wheat Cultures Under Different Treatments

**Table 3** lists some fermentation products related to MTFS from the culture extracts of the three groups of samples as detected by GC-MS. The results showed that the three group cultures mostly consisted of *N*-Hydroxymethylacetamide (retention time, 7.4 min), which accounted for 47, 30, and 65% in Groups D, E, and F. In particular, this study focused on pyridine materials such as 1-[2-Diethylaminoethyl]-2,3,4,5,6,7-hexahydro-4-oxo-1H-cyclopenta[b] pyridine (retention time, 10.95 min) that was detected at 2.28 and 2.23% by mass in Groups D and E, respectively, however, this was not detected in the cultures produced at the lower temperature used in treatment of samples in Group F.

**Table 3:** The GC-MS results of MTFS from the culture extracts of the three groups.

RT	Compound name	Molecular formula	CAS number	Area % D	Area % E	Area % F
5.64	Butanoic acid, 2,3-dimethyl-, methyl ester	C_7_H_14_O_2_	30540-29-5	17.22	14.78	-
5.74	Butanoic acid, ethyl ester	C_6_H_12_O_2_	105-54-4	3.30	2.88	-
6.31	Octanoic acid, 3,4-dimethylphenyl ester	C_16_H_24_O_2_	855371-61-8	0.82	1.25	-
6.83	Allyl(methoxy)dimethylsilane	C_6_H_14_OSi	18269-47-1	0.76	-	-
7.40	N-Hydroxymethylacetamide	C_3_H_7_NO_2_	625-51-4	47.09	30.26	65.48
7.63	1H-Purin-6-ol	C_5_H_4_N_4_O	95121-06-5	6.66	20.49	-
8.92	Imidazole, 1,4,5-trimethyl	C_6_H_10_N_2_	20185-22-2	2.36	1.44	-
10.95	1-[2-Diethylaminoethyl]-2,3,4,5,6,7-hexahydro-4-oxo-1H- cyclopenta [b] pyridine	C_14_H_24_N_2_O	18121-17-0	2.28	2.23	-
11.28	Acetamide, N-(2-benzoyl-4-chlorophenyl)-2-(2-methylpiperidin-1-yl)-	C_21_H_23_ClN_2_O_2_	83132-27-8	0.76	0.64	-
15.88	3-Methyl-1,4-diazabicyclo[4.3.0]nonan-2,5-dione, N-acetyl	C_10_H_14_N_2_O_3_	5248-89-5	0.80	-	-
18.01	N-Norvaline, n-propargyloxycarbonyl-, octyl ester	C_17_H_29_NO_4_	1228461-08-2	1.57	0.87	-
18.18	l-Valine, n-propargyloxycarbonyl-, tetradecyl ester	C_23_H_41_NO_4_	1252806-34-0	2.81	1.68	-
21.61	7,10-Hexadecadienoic acid, DMOX derivative	C_20_H_35_NO	56666-41-2	1.31	1.12	0.78
22.15	Octadecanoic acid, 2,3-dihydroxypropyl ester	C_21_H_42_O_4_	123-94-4	2.11	0.84	-
22.30	Artifact of roasted food iso-2	C_14_H_16_N_2_O_2_	3705-26-8	2.07	1.67	-
23.21	Hexadecanoic acid, 2-hydroxy-1-(hydroxymethyl) ethyl ester	C_19_H_38_O_4_	23470-00-0	1.06	-	-
24.69	n-Propyl-9.cis.,11.trans.- octadecadienoate	C_21_H_38_O_2_	276686-66-9	2.82	2.25	-

*CAS number, CAS (chemical abstracts service) register number.*

## Discussion

Spores represent intracellular dormancy of the *Bacillus* genus in response to environmental stress (nutrient deficiency, heat, UV, organic reagents, etc.) (Errington, 1993). In this research, *B. subtilis* was cultured at a high temperature of 55°C (Group A) and at a more normal temperature of 37°C (Group B). The spores were produced after cultivation for 12 h, and heat contributes to spore formation, resulting in the number of spores in Group A being significantly higher than that in Group B at the end of the culturation period. Moreover, the spore cortices in Group A are clearly thicker than those in Group B (**Figure 1**), which is a strategy of Bacilli spore for escaping damage (Popham et al., 1996).

**FIGURE 4:**
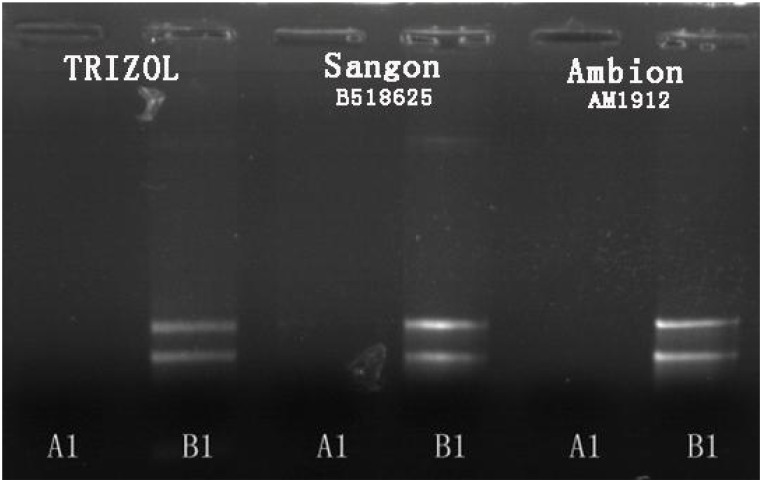
Agarose gel electrophoresis results for total RNA extraction from *B. subtilis* using three different methods.

Small-molecule acid-soluble spore proteins can bind onto DNA, which protects DNA from chemical and enzymatic cleavage. Moreover, a/β-type SASP—DNA interaction may also be promoted by the low free water content in the spore core (Setlow, 1992; Setlow et al., 1992). Up-regulated expressions of *sspA* and *sspC* and the low free water content in the spore core in Group A may be the reasons why the conventional method of RNA extraction fails for cells cultured at high temperature. In fact, several methods of total RNA extraction were conducted in this study: (i) the common TRIzol method, (ii) by using a Fungi Total RNA Isolation Kit (B518659, Sangon Biotech), and (iii) by using an RNAqueous^®^ Total RNA Isolation Kit (AM1912, Ambion). The latter two were aimed at thicker cytoderm and endogenous nuclease, respectively. The total RNA of *B. subtilis* in Group B was readily obtained using these methods, but Group A samples failed to produce results every time (see **Figure 4**).

**FIGURE 5:**
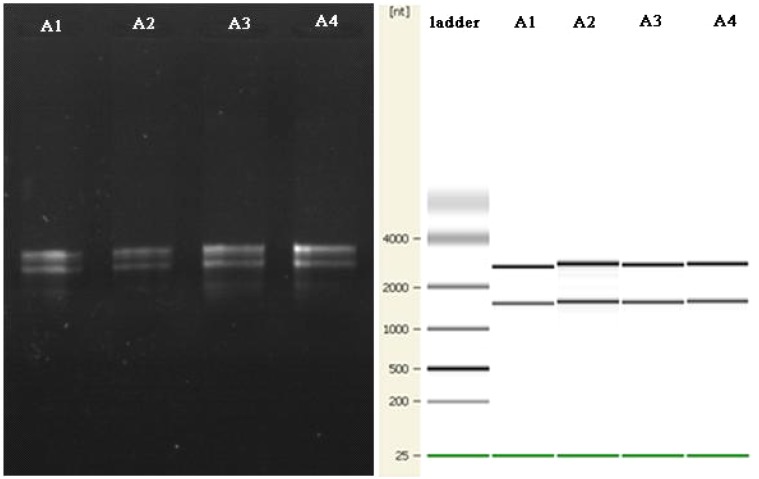
The agarose gel electrophoresis results for total RNA extraction from *B. subtilis* using the modified CTAB method are on the left. The quality control results for the Agilent 2100 are shown on the right.

There are reports of a modified CTAB method for total RNA extraction from plant leaves (Zhao et al., 2012; Lan et al., 2013; Morante-Carriel et al., 2014). So we tried a new way using a mixed CTAB solution for total RNA extraction. The method involves a combination of CTAB, lysozyme and proteinase K to destroy the cell walls and membrane proteins, and this is followed by an RNA separation phase where various chemicals are used together to release RNA and prevent its degradation. CTAB can selectively precipitate nucleic acids as well as dissolve membranes owing to its surfactant properties (Murray and Thompson, 1980). PVP and β-mercaptoethanol can prevent the loss and degradation of the RNA in the extraction process (Gehrig et al., 2000). LiCl can be used to make RNA precipitate specifically in the purification stage (Morante-Carriel et al., 2014). After much endeavor, the total RNA of *B. subtilis* in Group A was successfully obtained by using our modified CTAB method (**Figure 5**).

Many of the DEGs relate to tRNA-coding genes (**Figure 2**). Further analysis showed that they relate to tRNA of all kinds of amino acids — there is no preference for one, or one kind, of amino acid. This may be related to the synthesis of a variety of proteins or enzymes in the high temperature conditions. Wu and Xu (2012) reported that after culturing at high temperature the DEGs of *B. licheniformis* also exhibited the same result.

Regardless of whether we focus on the number of DEGs (**Figure 2**) or the number of *n*-fold changes in the DEGs (**Figure 3**), the results show that a noteworthy part of the DEGs are closely related to the formation of spores. Moreover, the up-regulated genes *pdaB, sspA*, and *sspC* are all related to the promotion of sporulation while the down-regulated genes *rapA* and *cotG* are related to the inhibition of sporulation. The details are as follows: PdaB mutation can cause a complete lack of muramic-δ-lactam which is a structural component of the peptidoglycan in the spore cortices (Warth and Strominger, 1972; Fukushima et al., 2004). This indicates that the up-regulated expression of *pdaB* promotes the formation of spore cortices in Group A and the TEM results seem to confirm this (**Figure 1**). Specificity lipoprotein produced by the encoding of *sspA* and *sspC* can help spore DNA resist UV and heat damage (Mason and Setlow, 1987). Protein phosphatase RapA acts specifically at the phosphotransferase Spo0F, which acts within a complex phosphorelay system important for activation of the master regulator Spo0A by its phosphorylation. At a certain threshold level, the phosphorylated Spo0A activates cellular entry into the sporulation process. The phosphatase (RapA) function is to drain the phosphorelay, reduce Spo0AP levels, and prevent sporulation. Thus down-regulation of *rapA* expression would result in a more efficient induction of sporulation (Perego et al., 1994). Saggese et al. (2014) proved that CotG exerts a negative regulation effect on the assembly of at least three spore coat proteins.

**FIGURE 6:**
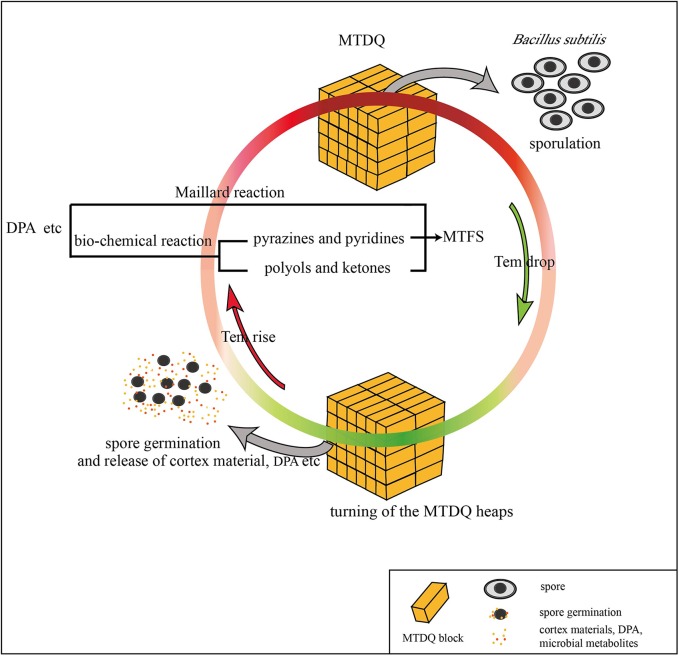
A possible way in which DPA released from the spore of *B. subtilis* may be connected to MTFS formation.

In this research, the different expression of genes related to sporulation and a thicker spore cortex appeared in *B. subtilis* under high-temperature culture conditions which may be responsible for protecting the bacteria from heat stress: however, is there a connection between the sporulation and the MTFS formation? Considering that the MTFS formed during high-temperature fermentation and the bacterial sporulation is promoted by higher temperatures, it is not groundless to suppose that the two phenomena are connected. In addition, the following research and analysis also show the similar associations. When PdaB transforms MurNAc into muramic-δ-lactam amino hexose, acetylated alanine and acetylated acetic acid are also generated as by-products (Warth and Strominger, 1972), which appear in the list of MTFS constituents (Li et al., 2014). Other, more persuasive, evidence is also associated with *pdaB*. Fukushima et al. (2004) reported that deletion mutation of *pdaB* decreases the spores’ dipicolinic acid (DPA) content to only 1.2% of that of the wild type. This suggests that there is a certain connection between DPA formation and the function of PdaB. DPA is an important part of the spore, and forms a large proportion of the dry weight of the spores. It also plays an essential role in spore heat resistance (Errington, 1993). One key point is that DPA contains a pyridine ring, and is therefore a potential precursor for pyridine derivatives (Li et al., 2012, 2014). Zhuang (2005) found that Maotai-style liquors have a higher content of several heterocyclic compounds including the pyridine and 3-isobutyl pyridinithiazole. These kinds of heterocyclic nitrogen compounds are ingredients in the MTFS (Fan et al., 2011; Li et al., 2014). Presumably, microbes proliferate rapidly during the gradually rising temperature stage of fermentation. As the temperature rises, the bacilli form spores to resist the heat. A large amount of cortex material (peptidoglycan, amino acids) and DPA is accumulated in the spores. When the heaps of MTDQ are turned over ready for a new round of fermentation, the spores will germinate as the MTDQ cools. At this time, the spore walls develop into cell walls as well as the cortex, and DPA will be released (Kay and Warren, 1968; Setlow, 2014). These materials released into the MTDQ may act directly as a kind of MTFS or they may become various pyridine derivatives. They may also become reactants in the Maillard reactions taking place in the next round of rising-temperature fermentation (Pripis-Nicolau et al., 2000; Zhang et al., 2013). This process is demonstrated graphically in **Figure 6**. In fact, the DPA content is significantly higher in spores produced during high-temperature culturing than in spores produced at lower temperatures (please see section “The DPA Content of Bacillus Spores Under the Two Different Culture Conditions”). Considering that the heat-resistant *Bacillus* spp. of MTDQ, such as *B. amyloliquefaciens* and *B. licheniformis* (Li et al., 2014) are the dominant bacteria, thus a contribution of sporulation in the Maotai production might be really high. This supports the evidence for the hypothesis, that the formation and release of the spores’ DPA via six or more rounds of fermentation featuring from low- to high-temperature may be one of the sources of heterocyclic nitrogen compounds in the MTFS. Many researchers adopt the view that MTFS precursors are metabolites produced by microbial fermentation (Zhu et al., 2010; Wu and Xu, 2012). Therefore, analyzing the relevant metabolic pathways of the DEGs can help to explain the mechanism of MTFS formation; however, the results show that only two DEGs (*kbl* and *ripA*) are related to material metabolism. It is notable that the products of their catalytic function, aminobutyric acid and dihydroxy butanone phosphoric acid, are common ingredients in MTFS (Zhuang et al., 1999; Lin et al., 2013; Li et al., 2014). This indicates that the bacteria can up-regulate the expression of some genes in high temperature conditions which promotes the formation of some special metabolites. These may become part of the MTFS or be their precursors.

To validate the hypothesis further, six rounds of wheat fermentation featuring low- to high-temperature conditions were conducted. Analysis of GC-MS output showed that one pyridine-like compound which could potentially originate from the spores’ DPA was detected in both Groups D and E, with a minor relative increase in the amount thereof in Group D, but low-temperature cultures with these bacteria (in Group F) did not contain this pyridine-like compound (see **Table 3**). This suggests that MTFS (as pyridine) comes from the high-temperature fermentation of wheat and microbes (*B. subtilis*, in our experiments). It has been known that flavor compounds in Maotai liquor are complicated and more than 1,000 flavors have been detected (Ji et al., 2007); however, the Maotai-style liquor usually contains 1/100,000 to 1/1,000,000 proportion of the aroma components, and the liquor fragrance depends on these compounds present even in such low concentrations (Mi and Zhang, 2012), and minor changes in composition (such as pyridine-like compound) are enough to affect the aroma and taste of the liquor significantly (Li et al., 2012; Chen et al., 2013; Sun et al., 2017). In addition, only two aromatic compounds produced in the cultures of Group F were detected (wheat fermentation with *B. subtilis* in low-temperature fermentation). Many aromatic compounds were present in Groups D and E, Group D had a higher concentration of some compounds and Group E had a higher concentration of others, which is hard to explain due to the complex chemical processes and re-catabolism by the bacteria. It is noteworthy that several compounds including {Allyl(methoxy)dimethylsilane}, {3-Methyl-1,4-diazabicyclo[4.3.0]nonan- 2,5-dione, N-acetyl} and {Hexadecanoic acid, 2-hydroxy-1-(hydroxymethyl)ethyl ester} appeared only in Group D, which suggested that these compounds are potentially related to the role of *B. subtilis*.

It should be emphasized that the Maillard reaction is one of the important sources of MTFS during the high-temperature fermentation of MTDQ (Lian, 1995; Zhuang et al., 1999; Liu et al., 2007; Fan and Xu, 2012). The cortex materials, DPA, microbial metabolites, and other compounds from wheat may act as reactants in the Maillard reactions and generate complex, diverse products in Group D samples.

## Conclusion

The DEGs of *B. subtilis* under high-temperature solid fermentation in WEBP medium are mainly related to the formation of spores. More DPA is accumulated in spores during the high-temperature fermentation stage and then released as the spores germinated during the start of the next round of fermentation. Although the exact mechanism of the process remains unknown, it might be possible that, after several cycles, DPA or its derivatives were transformed into the characteristic MFTS by a combination of the Maillard reaction and bacterial fermentation. In our experiments, a modified CTAB method was designed and successfully applied to extract the total RNA of *B. subtilis* under high-temperature fermentation, and could be used as an approach for RNA extraction of other main bacilli from MTDQ for the further gene function research.

## Author Contributions

BL and WW wrote the main manuscript text. BL and WW designed the experiments. RL, YS, and WW carried out the experiments. All authors reviewed the manuscript.

## Conflict of Interest Statement

The authors declare that the research was conducted in the absence of any commercial or financial relationships that could be construed as a potential conflict of interest.

## Footnotes

1 http://www.ncbi.nlm.nih.gov/geo/

## Supplementary Material

FIGURE S1 The counting of bacterial spores in Group A and B at different temperatures and stages.
