# Supplementary figures and images for: The Potential Correlation Between Bacterial Sporulation and the Characteristic Flavor of Chinese Maotai Liquor
Source: Front Microbiol. 2018 Jul 2;9:1435. doi: 10.3389/fmicb.2018.01435 (PMC6037195; doi:10.3389/fmicb.2018.01435)

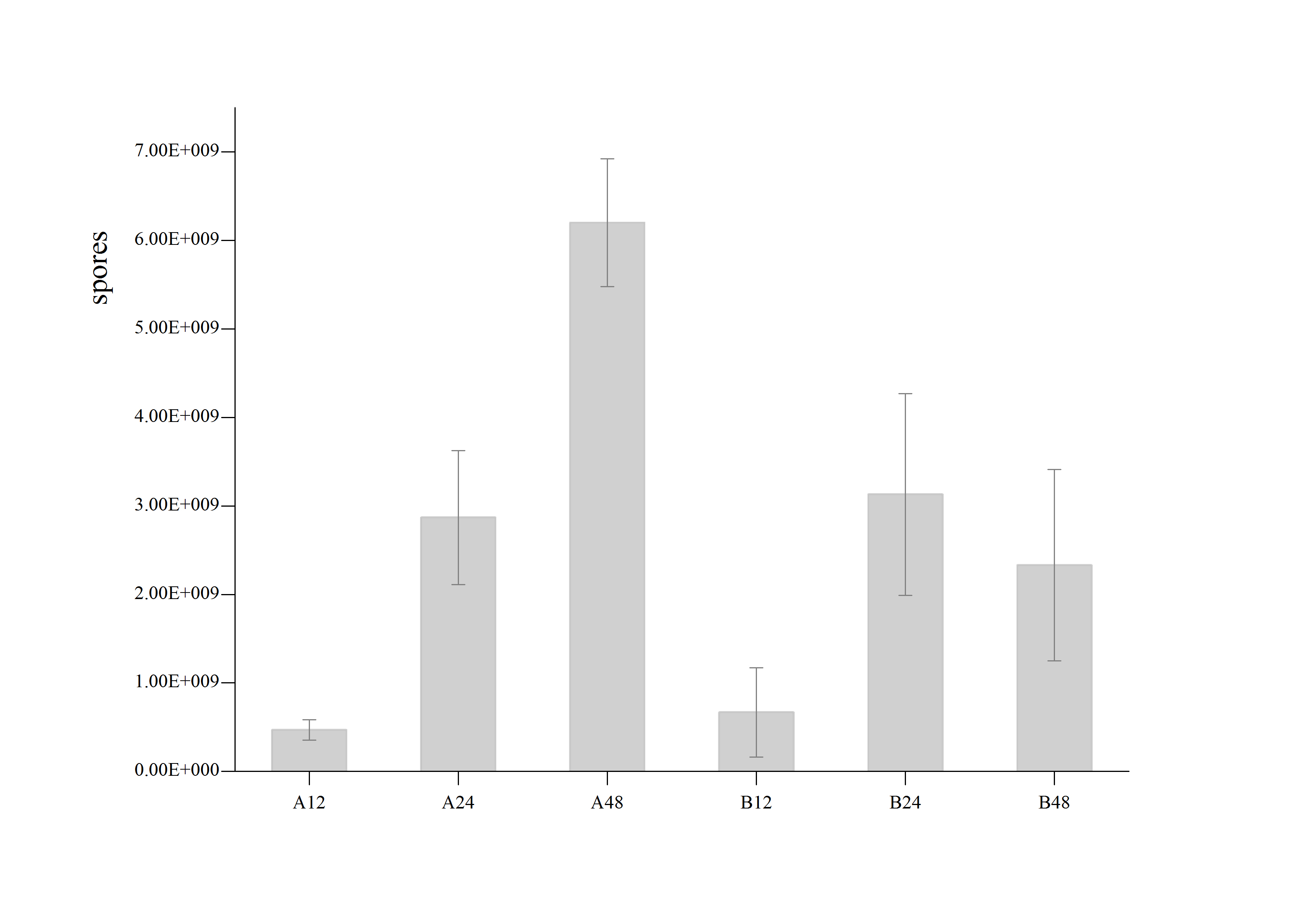

Supplement: FIGURE S1 — The counting of bacterial spores in Group A and B at different temperatures and stages. [file Image_1.JPEG]
